# Characterization of a Small Cysteine-Rich Secreted Effector, TcSCP_9014, in *Tilletia controversa*

**DOI:** 10.3390/plants13111523

**Published:** 2024-05-31

**Authors:** Zhenzhen Du, Han Weng, Huanyu Jia, Bin Zhang, Boming Wu, Wanquan Chen, Taiguo Liu, Li Gao

**Affiliations:** 1 State Key Laboratory for Biology of Plant Diseases and Insect Pests, Institute of Plant Protection, Chinese Academy of Agricultural Sciences, Beijing 100193, China; 2 College of Plant Protection, China Agricultural University, Beijing 100193, China; 3 Institute of Plant Protection, Xinjiang Academy of Agricultural Sciences, Key Laboratory of Integrated Pest Management on Crop in Northwestern Oasis, Ministry of China, Scientific Observing and Experimental Station of Korla, Ministry of Agriculture, Urumqi 830091, China

**Keywords:** wheat dwarf bunt, *Tilletia controversa*, small cysteine-rich secreted proteins, virulence effectors, plant immunity

## Abstract

*Tilletia controversa* J. G. Kühn is the causal agent of wheat dwarf bunt (DB), a destructive disease causing tremendous economic losses. Small cysteine-rich secreted proteins (SCPs) of plant fungi are crucial in modulating host immunity and promoting infection. Little is known about the virulence effectors of *T. controversa*. Here, we characterized TcSCP_9014, a novel effector of SCPs, in *T. controversa* which suppressed programmed cell death triggered by BAX without relying on its signal peptide (SP). The SP in the N-terminus of TcSCP_9014 was functional in the secretory process. Live-cell imaging in the epidermal cells of *Nicothiana benthamiana* suggested that TcSCP_9014 localized to the plasma membrane, cytoplasm, and nucleus. Furthermore, yeast cDNA library screening was performed to obtain the interacting proteins in wheat. Yeast two-hybrid and BiFC assays were applied to validate the interaction of TcSCP_9014 with TaMTAN and TaGAPDH. Our work revealed that the novel effector TcSCP_9014 is vital in modulating plant immunity, which opens up new avenues for plant-pathogen interactions in the *T. controversa* infection process.

## 1. Introduction

*Tilletia controversa* J. G. Kühn, belonging to Basidiomycotina [1], is an important quarantine pathogen worldwide. Wheat dwarf bunt (DB), caused by *T. controversa*, is currently distributed in Asia, Europe, Africa, North America, and South America, and many countries have developed strict restrictions on imported wheat infected by DB [2,3,4]. Notably, the teliospores of *T. controversa* dispersed in soil are able to survive for up to 10 years [5], posing great difficulties in the prevention and control of DB, which mainly spreads by way of soil [6,7]. The yield loss caused by DB in an epidemic year is as high as 75–80% [1,6,8], and the grains of infected wheat are replaced by bunt balls (sori) filled with teliospores. In addition to the devastating economic impact, DB also seriously affects the quality of flour because of the presence of trimethylamine in the teliospores, leading to a fishy smell. Flour processed from infected wheat grains containing pathogenic spores poses a grave threat to the health of humans and animals [2].

In the long-term co-evolution process of host plants and pathogens, like in an arms race, plants have evolved multilayer innate immunity and pathogens have developed various strategies to improve their virulence. Firstly, pathogen-associated molecular patterns (PAMPs) are specifically recognized by pattern recognition receptors (PRRs) on plant cell membranes, causing an early response of pattern-triggered immunity (PTI) [9,10]. To interfere with the PTI reaction of plants, pathogens have successfully evolved and delivered effectors to hosts. Through effectors, pathogens can surmount the first defense of the plant immune system. Meanwhile, plants further produce receptors of resistance proteins (R proteins) specifically recognizing effectors. Then, stronger effector-triggered immunity (ETI) [9,10,11] is activated. In plants, effectors are recognized by R proteins directly or indirectly. In direct recognition, R proteins and effectors are directly recognized to activate the ETI. In indirect recognition, R proteins are guard proteins, and effectors target the ‘guardee’ guarded by the R proteins (the guard) in the host. When the ‘guardee’ protein is bound and modified by effector, the corresponding R protein senses it and initiates immune responses [12]. The recognition of effectors and R proteins usually causes programmed cell death (PCD), known as the necrosis reaction (HR) [13]. The PCD reaction in plants plays a crucial role in stopping the spread of pathogenic fungi [14]. This is the well-known “zig-zag” model used to describe the arms race in the co-evolution of plants and pathogens [9,15,16].

Plant-pathogenic fungi are the primary agents of many crop diseases [17]. Although they are diverse, they share a common plant colonization strategy of modulating the defense systems of hosts by secreting effectors [18,19,20]. Usually, effectors can be secreted to the extracellular and cytoplasmic spaces of a host cell [19]. Previous studies found that many fungal effectors belong to the small secreted cysteine-rich proteins (SCPs) [13,21]. Cysteine residues may be involved in forming disulfide bond bridges, thus contributing to the stability of effectors and assisting pathogenic fungi to survive in harsh environments to promote infections in hosts [13,22,23]. Effectors also exhibit two other characteristics: less than 300 amino acids and great differences in sequences [24]. Typically, some of them contain a signal peptide (SP) structure in the N-terminus and are secreted through the classical pathway from the endoplasmic reticulum to the Golgi apparatus. However, with the discovery of an increasing number of effectors, a significant subset has been identified that lack signal peptides (SPs). These effectors are secreted through unconventional pathways, which include vesicular, non-vesicular, and vacuolar-mediated mechanisms. [25,26,27].

At present, investigations into effectors in plant pathogenic fungi are abundant. For example, in *Magnaporthe oryzae*, Li et al. [28] reported that of 3315 effectors, 509 proteins (15.4%) contain an SP in the N-terminus. In contrast, 2806 proteins (84.6%) do not. 416 proteins are functional in secretion, while 93 proteins are not. From 18 randomly selected effectors, 16 of them, such as G4MVY9, G4NAI7, and Q2KEU7 were found to inhibit PCD induced by Bax through agroinfiltration-mediated transient expression in *Nicothiana benthamiana*. In *Plasmodiophora brassicae*, 55 candidate effectors in 518 secretary proteins were found to suppress BAX-induced cell death. The subcellular localization of five candidate effectors with the RXLR motif showed that the fluorescence signal was distributed in the cytoplasm and cytomembrane [29]. According to the description by Li et al. [30], two effector candidates, CSEP0139 and CSEP0182, of *Blumeria graminis* f. sp. *hordei* (*Bgh*) were identified. They both contain an SP that is functional in the secretory process and suppresses cell death triggered by BAX. The subcellular localization revealed that they both localized to the cytosol and nucleus when overexpressed in epidermal cells of *N. benthamiana*. In the smut fungi, the genome of *Tilletia horrida* and 597 secreted proteins were demonstrated [31]. Similar to Bax, two putative effectors, smut_5844 and smut_2964, can induce necrosis in *N. benthamiana*. The functional validation of the SP indicated their secretion function [31].

There is currently limited information about the virulence effectors of *T. controversa*. Based on genome sequencing by Nguyen et al. [32] and the characteristics of the SCPs mentioned above, the prediction of effectors was carried out through bioinformatics, and specific primers were designed to clone the gene encoding *T. controversa* effectors. We successfully cloned the gene of TcSCP_9014, a typical SCP that is small, rich in cysteine, contains an SP, and does not have a known conserved domain. Therefore, it may be a novel effector secreted by *T. controversa* to promote infection.

In this study, TcSCP_9014 was successfully characterized. The suppression detection of PCD triggered by Bax, a functional analysis of secretion by SP, subcellular localization observations, and cNDA library screening were conducted. In parallel, the interaction between TcSCP_9014 and two host proteins was validated to explain whether these aspects are involved in manipulating plant immunity and promoting the virulence of *T. controversa*.

## 2. Results

### 2.1. Bioinformatics Analysis of TcSCP_9014

**Figure 1:**
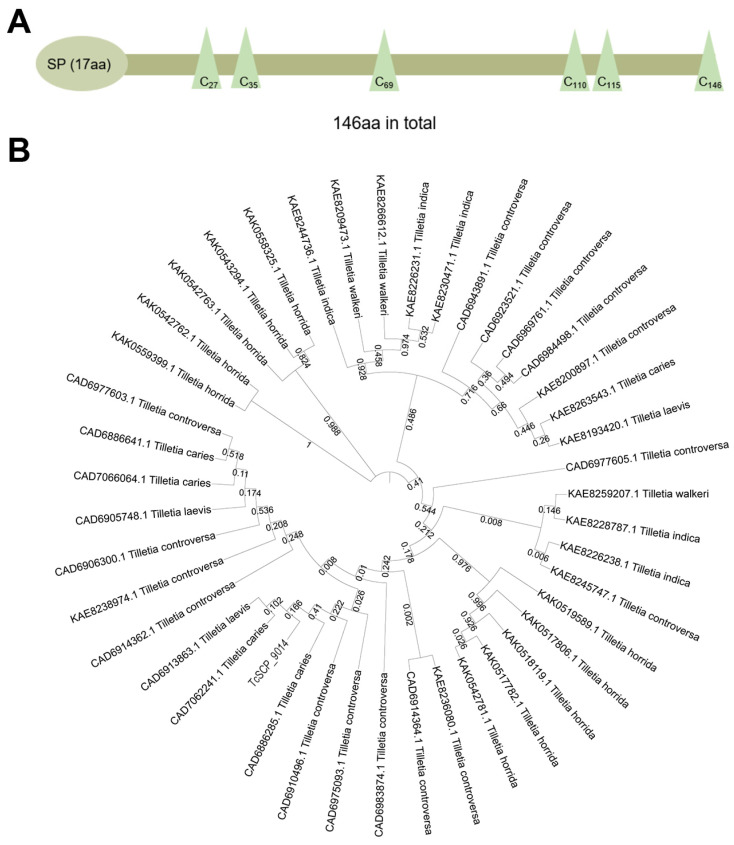
Protein structural characteristics and phylogenetic analysis of TcSCP_9014. (**A**) Protein structural characteristics of TcSCP_9014. TcSCP_9014 contains a putative SP (17aa) in the N-terminus and six cysteine residues (C_27_, C_35_, C_69_, C_110_, C_115_, C_146_). (**B**) Phylogenetic tree of TcSCP_9014 and its homologs from five related *Tillitia* species generated by MEGA 7.0 software.

According to a report on the genome sequencing of *T. controversa* by Nguyen et al. [32], we designed specific primers. Then, the gene of a novel effector TcSCP_9014, which consists of 441 nucleic acids and encodes a 146-amino-acid (aa) protein, was successfully cloned. Through SignalP4.0, it was predicted to contain an SP with 17aa in the N-terminus (Figure 1A). Meanwhile, six cysteine residues were distributed in the amino acid sites of C_27_, C_35_, C_69_, C_110,_ C_115_, and C_146_ (Figure 1A). An analysis by online software also showed that TcSCP_9014 was localized to the cytoplasm. In addition, conserved motifs and transmembrane domains were not found in TcSCP_9014. Phylogenetic analysis revealed the relationship between TcSCP_9014 and its homologous proteins in closely related species of *Tilletia*, including *Tilletia laevis*, *Tilletia caries*, *Tilletia indica*, *Tilletia walkeri*, and *Tilletia horrida* (Figure 1B). Although there are homologous proteins of TcSCP_9014 in *T. controversa*, the most closely related proteins to TcSCP_9014 are from *T. laevis* and *T. caries*, rather than from *T. controversa* itself (Figure 1B).

### 2.2. Suppression of PCD by TcSCP_9014

**Figure 2:**
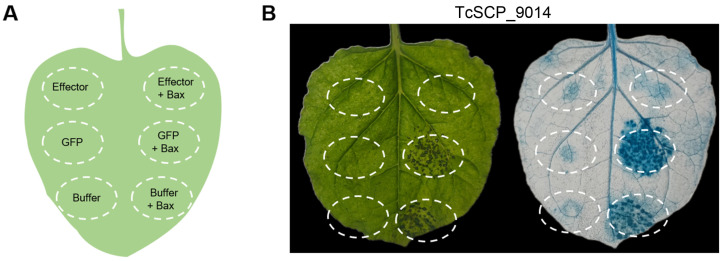
Suppression of Bax-triggered PCD by TcSCP_9014. (**A**) Schematic representation of injection in *N. benthamiana* leaves. The gene sequence of *TcSCP_9014* without the SP and Bax were introduced into the pGR107 vector and transformed into *A. tumefaciens*. Transient expression was conducted by agro-infiltration. We inoculated *A. tumefaciens* carrying pGR107-TcSCP_9014, pGR107-GFP, and buffer on both the left and right sides of *N. benthamiana* leaves, and a challenging inoculation of pGR107-BAX was performed on the right sides of the leaves after 24 h. pGR107-GFP and buffer were used as negative controls. (**B**) *N. benthamiana* leaves were photographed before and after trypan blue staining. The injection contents inside the dotted circles in the *N. benthamiana* leaves were those indicated in Figure 2A.

The transient expression of TcSCP_9014 in *N. benthamiana* was achieved by agro-infiltration assays. *A. tumefaciens* carrying pGR107-BAX was inoculated 24 h after the injection of *A. tumefaciens* carrying pGR107-TcSCP_9014 to detect whether TcSCP_9014 suppressed the PCD induced by Bax (Figure 2A). pGR107-GFP and injection buffer were used as negative controls (Figure 2A). Bax is an apoptosis factor, an activator of PCD derived from mouse [33]. Through it, rapid high-throughput screening of effectors in various pathogenic fungi has been realized. After the inoculation of pGR107-BAX, the phenotypes of the leaves of *N. benthamiana* implied that TcSCP_9014 suppressed Bax-triggered cell death (Figure 2B), while the injection areas of pGR107-GFP and buffer showed necrosis. The injected leaves of *N. benthamiana* showing cell death were stained with trypan blue, which confirmed the results of PCD suppression by TcSCP_9014 (Figure 2B).

### 2.3. TcSCP_9014 Contains a Functional SP

**Figure 3:**
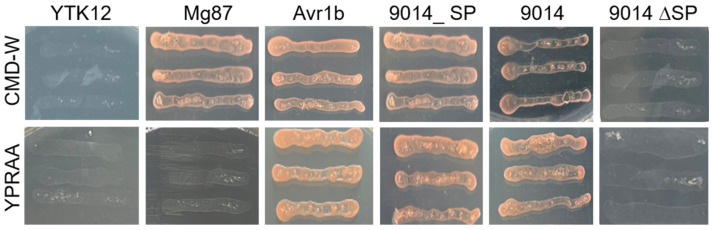
Functional detection of the SP. pSUC2 recombinant vectors were constructed with the SP sequence (9014_SP), the full-length sequence (9014), and the full-length sequence without the SP (9014∆SP) in TcSCP_9014, then yeast transformation was conducted. Yeast invertase secretion assay was implemented with CMD-W and YPRAA media. The growth of yeast transformants on both media suggested a secretory function of the SP in TcSCP_9014.

The prediction by SignalP4.0 software revealed that a typical SP structure was located in the N-terminus of TcSCP_9014. To detect whether the SP is functional in secretion, we cloned the sequences of SP, the full length, and the full length without the SP in TcSCP_9014. Subsequently, the corresponding pSUC2 recombinant vectors were constructed and transformed into the yeast YTK12 strain. The SP sequence of the effector Avr1b from *Oomycete* and the first 25-amino-acid sequence of Mg87 in *M. oryzae* were used as the positive and negative controls, respectively. CMD-W and YPRAA screening media were applied. We found that yeast transformants expressing the SP (9014_SP), the full length of TcSCP_9014 (9014), and the positive control Avr1b grew on both media, and the negative control Mg87 grew only on the CMD-W medium. In contrast, the yeast YTK12 and the full length without the SP (9014∆SP) could not grow on either medium (Figure 3), indicating that TcSCP_9014 contains a functional SP in the N-terminus.

### 2.4. Subcellular Localization in N. benthamiana Epidermal Cells

**Figure 4:**
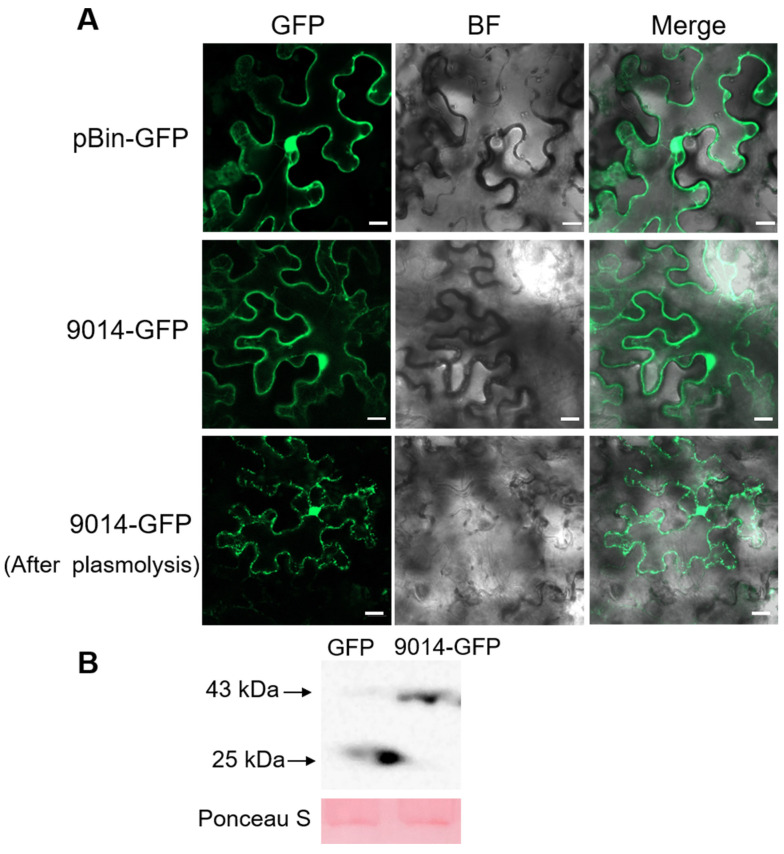
Subcellular localization of TcSCP_9014. (**A**) The gene sequence of TcSCP_9014 without the SP was fused in the pBin-GFP vector and transformed into *A. tumefaciens*. Transient expression was applied. After 2–3 days, the fluorescence signal of GFP was tracked to determine the subcellular localization of 9014-GFP in *N. benthamiana* epidermal cells. Scale: 10 μm. Plasmolysis was also performed to visualize the localization of GFP signals. (**B**) GFP and 9014-GFP expressions in *N. benthamiana leaves* were detected by Western blotting.

Although we predicted the subcellular localization of TcSCP_9014 with the online software PSORT (Version 3.0.3), to further determine its localization information, we constructed the pBin-GFP recombinant vector (9014-GFP) and used the transient expression mediated by *A. tumefaciens* to observe the localization of 9014-GFP in *N. benthamiana* epidermal cells under a laser confocal microscopy. In parallel, we conducted plasmolysis assay of *N. benthamiana* epidermal cells using 0.5M NaCl according to Chen et al. [34]. The results suggested that the GFP gene was expressed successfully (Figure 4A), and the fluorescence signal of 9014-GFP was detected in the plasma membrane, the cytoplasm, and a tiny amount of the nucleus before and after plasmolysis (Figure 4A). Furthermore, Western blotting confirmed that after GFP fusion, the protein 9014-GFP remained intact and was not degraded to free GFP (Figure 4B; Supplementary Figure S1). Therefore, TcSCP_9014 was localized to the plasma membrane, cytoplasm, and nucleus. 

### 2.5. Screening and Validation of Interacting Proteins 

Based on the subcellular localization results, the effector TcSCP_9014 was mainly distributed in the plasma membrane and cytoplasm, while only a tiny GFP signal was detected in the nucleus. Therefore, we chose the split-ubiquitin membrane system, which is amenable to screening for the interaction of membrane proteins and cytoplasmic proteins [35]. If two different proteins interact, then the proteolytic cleavage of protein fusion will be detected, releasing a transcription factor to ultimately activate the reporter gene in the nucleus [35]. To screen the cDNA library constructed from infected wheat spike samples, we first constructed the recombinant vector pDHB1-TcSCP_9014, used as a bait vector. Then, the functional validation was performed. The positive control was pDHB1-Large T and pDSL-p53, and negative control was pDHB1-Large T and pPR3-N. The two tested groups were pDHB1-*TcSCP_9014* with pOst1-NubI, and pDHB1-TcSCP_9014 with pPR3-N. After yeast transformation, the growth of the transformants on the three screening media DDO, TDO, and QDO was observed.

We found that both the transformants of the positive control and pDHB1-*TcSCP_9014* with pOst1-NubI grew normally on the three screening media (Supplementary Figure S2A,C), while transformants of negative group and pDHB1-*TcSCP_9014* with pPR3-N grew only on DDO medium (Supplementary Figure S2B,D), indicating that the bait vector was normally expressed in yeast and showed no interaction with empty library plasmids. According to the testing results for the 3-AT concentration (Supplementary Table S2), QDO plates with 15 mM 3-AT, without colony growth, can be used for cDNA library screening.

**Figure 5:**
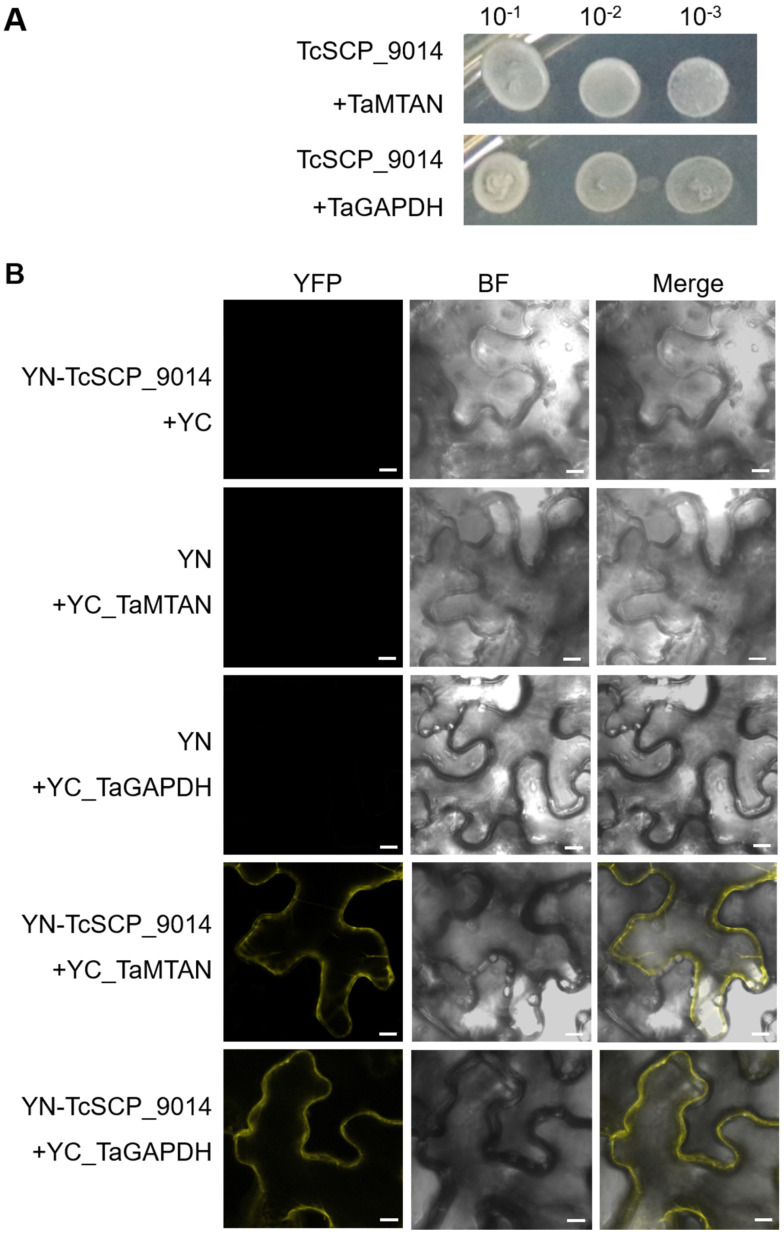
Validation of the interactions between TcSCP_9014 and two host proteins. (**A**) One-on-one yeast two-hybrid assay to validate the interactions between TcSCP_9014 and host proteins of TaMTAN, TaGAPDH. After the plasmids extraction of the interacting proteins and co-transformation with bait plasmids pDHB1-TcSCP_9014 into yeast of the NYM51 strain, the growth of positive colonies in QDO/15 mM 3-AT medium suggested interactions between TcSCP_9014 and the two host proteins. (**B**) BiFC assay to validate the interactions between TcSCP_9014 and host proteins of TaMTAN, TaGAPDH. After the construction of recombinant vectors of p2YC (cYFP) and p2YN (nYFP), *A. tumefaciens* transformation and agro-infiltration were carried out. Observation of the YFP signal under a laser confocal microscope was performed after 2–3 days. Scale: 10 μm.

Colonies expressing interacting proteins were obtained by transforming the cDNA yeast library into competent cells expressing bait plasmids and screening them on QDO plates with 15 mM 3-AT. PCR assay was used to detect the positive colonies, and PCR products showing target bands of more than 1000 bp were sent for sequencing. After BlastX, the information on the interacting proteins in the host was clear. We selected two candidate proteins with annotations of 5-methylthioadenosine-like S-adenosylhomocysteine nucleosidase 2-like (TaMTAN) and glyceraldehyde-3-phosphate dehydrogenase (TaGAPDH). For colonies expressing the two candidate interacting proteins, we conducted plasmid extraction; then, yeast transformations of bait plasmids and one kind of prey plasmids of the interacting protein were performed for one-on-one yeast two-hybrid identification (Figure 5A). Further validation of the interactions in TcSCP_9014 with TaMTAN and TaGAPDH was conducted through Bimolecular fluorescence complementary (BiFC) assay (Figure 5B).

## 3. Discussion

The standard of SCPs for plant fungal effectors has been widely applied [25]. Currently, many fungi effectors with SCPs have been characterized, including effectors of *T. horrida* [31], *Verticillium dahlia* [36], *Rhizoctonia solani* [37], and *Bipolaris sorokiniana* [38]. In this study, we successfully cloned the encoding gene of effector TcSCP_9014 in the secretory group of *T. controversa*. A bioinformatic analysis showed a typical SP structure in the N-terminus (Figure 1A), which is consistent with the sequence characterization of the effectors of filamentous fungi reported by Lovelace et al. [21]. Although many SCP-type filamentous fungal effectors contain specific motifs, such as RXLR and CRN, proteins without specific candidate motifs have also been identified from different pathogens [39,40,41]. Therefore, TcSCP_9014, without any conserved motifs, may represent a novel effector evolved by *T. controversa* to better to modulate host immunity in an unreported manner.

*A. tumefaciens*-mediated infiltration in *N. benthamiana* is an efficient way to quickly identify the function of effectors in the suppression of plant innate immunity. Bax is an apoptosis-promoting mouse protein. Although the homologous protein of BAX may not exist in plants, it functions in plants and can induce PCD [42]. Through the screening system in *N. benthamiana*, we found that TcSCP_9014 suppressed BAX-induced PCD (Figure 2B). The suppression of a plant’s innate immunity is crucial for pathogens to invade plant cells successfully [43]. Therefore, TcSCP_9014 was regarded to be involved in manipulating plant immunity by suppressing cell death during the infection of wheat cells with *T. controversa*. The identification of TcSCP_9014 was based on the removal of the SP sequence, suggesting that the SP is unnecessary for suppressing the cell death induced by Bax. However, in the secretion process, the SP is essential. In summary, *TcSCP_9014* is secreted by *T. controversa* to suppress PCD in host cells in order to promote virulence. 

The subcellular localization of TcSCP_9014 was observed in the epidermal cells of *N. benthamiana*, and it was localized to the plasma membrane, cytoplasm, and nucleus (Figure 4), implying that the function of TcSCP_9014 in manipulating plant immunity was activated in these cell structures. According to the subcellular localization result and the prediction of the transmembrane domain of the TcSCP_9014, cDNA library screening was performed with bait vector pDHB1-TcSCP_9014 through the split-ubiquitin membrane system. Thus, we obtained the interacting proteins of TcSCP_9014 in the wheat host. Through yeast two-hybrid and BiFC assays, we verified the interaction of TcSCP_9014 with TaMTAN and TaGAPDH (Figure 5A,B). Chen et al. [44] reported that MTAN, an enzyme of many critical biological processes, is an attractive antibacterial drug target in mammals. GAPDH is a key enzyme in the glycolytic pathway and is vital in maintaining a normal state. For example, in *Arabidopsis*, there are four members in the GAPDH family, AtGAPC1, AtGAPC2, AtGAPCp1, and AtGAPCp2 [45]. They are essential in growth and development and in the balance of C/N in cells, and AtGAPCp is involved in the plant ABA signaling pathway [46,47]. Wagner et al. [48] found that the GAPDH (2–32 aa) peptide segment in human placental tissue shows antibacterial properties against pathogenic yeast (*Candida albicans*). An elucidation of the functions of the interacting proteins TaMTAN and TaGAPDH in wheat will help to enrich their antifungal roles. We will conduct functional analyses on these two interacting proteins in future work to better reveal the host resistance strategy against *T. controversa* and to provide valuable resources for breeding work.

## 4. Conclusions

Taken collectively, we characterized a novel SCP effector, TcSCP_9014, secreted by *T. controversa*. A functional analysis demonstrated that it could manipulate host immunity by suppressing PCD to facilitate the infection of *T. controversa*, a process that does not rely on its SP. The SP only functions in secretion processes. The subcellular localization observations revealed that TcSCP_9014 was localized to the plasma membrane, cytoplasm, and nucleus. Yeast cNDA library screening was performed, and the corresponding host interacting proteins were successfully obtained. The interaction of TcSCP_9014 with two wheat proteins, TaMTAN and TaGAPDH, which may act as R proteins in the host, was validated by yeast two-hybrid and BiFC assays. Our findings provide new insights into the infection strategy of *T. controversa* and the host resistance mechanism, as well as providing a valuable reference for efficient disease control measures.

## 5. Materials and Methods

### 5.1. Plant and Fungal Material

*N. benthamiana* was planted at 23 °C (±2 °C) for 4–6 weeks in a greenhouse with 16 h of light and 8 h of dark. The tested strain of *T. controversa* was kindly provided by Blair Goates, United States Department of Agriculture (USDA), Agricultural Research Service (ARS), Aberdeen, ID, USA. The teliospore suspension of *T. controversa* was adjusted to 10^6^/mL of OD_600_, and 220 μL of the suspension was spread and coated onto soil plates. The plates were placed in an artificial incubator for cultivation. The setting criteria of the incubator were as follows: 5 °C, 2 Lx light, and 50% relative humidity. After three weeks, germination was continuously observed under a microscope, and different growth stages of hyphae in *T. controversa* were collected.

### 5.2. Bioinformatics Analysis

The cleavage site of the SP in TcSCP_9014 was predicted through the online software tool SignalP4.1 (https://services.healthtech.dtu.dk/services/SignalP-4.1/, accessed on 15 October 2022). The phylogenetic tree of the effector TcSCP_9014 and its homologous protein was constructed through ClusterW in MEGA 7.0 software. The subcellular localization analysis was conducted through the online software tool PSORT (https://wolfpsort.hgc.jp, accessed on 15 October 2022). The transmembrane helical structure prediction was performed using Predictprotein (https://www.predictprotein.org/, accessed on 15 October 2022) and the Transmembrane Prediction server (https://sbcb.bioch.ox.ac.uk/TM_noj/TM_noj.html, accessed on 15 October 2022). The conserved motifs were analyzed using the InterPro database [49]. 

### 5.3. Construction of Recombinant Vectors PGR107 and pBin-GFP

RNA was extracted from different growth stages of *T. controversa* hyphae with Trizol reagent (Invitrogen, Carlsbad, CA, USA) according to the manufacturer’s instructions, and its quantity and quality were detected by a NanoDrop device (Denovix, DE, USA). Then, the first strand of cDNA was synthesized by M-MLV reverse transcriptase (Invitrogen, Carlsbad, CA, USA). The gene sequence of BAX with accession number L22472.1 (GenBank: https://www.ncbi.nlm.nih.gov/, accessed on 20 October 2022) was synthesized by Beijing Tsingke Biotech Co., Ltd. The specific primer (Supplementary Table S1) used for PGR107 and pBin-GFP recombinant vector construction was designed for the effector (without the SP) and for Bax used in the construction of the PGR107 recombinant vector. The enzyme restriction sites were *Sal*I and *Cla*I for PGR107, and *BamH*I and *EcoR*I for pBin-GFP. Next, the *TcSCP_9014* and *Bax* gene fragments and linearization vectors were fused through the ClonExpress Ultra One Step Cloning Kit (Vazyme, Nanjing, China) and they were transformed into DH5α competent cells. After PCR detection, the positive colonies were sent for sequencing to confirm whether the recombination was successful.

### 5.4. Transient Expression Assays and Suppression of PCD

The transient expression of genes mediated by *A. tumefaciens* in *N. benthamiana* was assessed according to Li et al. [30] to analyze whether the effector could suppress the cell death induced by Bax. *A. tumefaciens* carrying GFP and TcSCP_9014, as well as injection buffer, was injected into tobacco leaves. After 24 h, a challenge inoculation of *A. tumefaciens* carrying pGR107-BAX was conducted.

### 5.5. Trypan Blue Staining

Cell death phenotypes in *N. benthamiana* leaves were photographed after 5 days and further validated by trypan blue staining. In detail, the process was as described by Li et al. [30]. Trypan blue staining solution and ethanol were mixed in equal volume, and the leaves were boiled with the mixture for 5 min. After overnight staining and de-staining, photos were taken.

### 5.6. Secretory Analysis of SP

The cleavage site of the SP was predicted through SignalP4.0. The vector pSUC2 was digested with the restriction enzyme of *EcoR*I and *Xho*I, and the sequences of SP, the full length, and the full length without the SP in TcSCP_9014 with homologous arms were cloned with specific primers (Supplementary Table S1). Then, the cloned sequences were introduced to the pSUC2 vector. The transformation of yeast YTK12 competent cells was applied subsequently. Positive colonies were randomly selected and transferred to SD/Trp liquid medium. They were cultured at 28 °C in an incubator for 2 days. A volume of 5 μL of the mixture was coated onto CMD-W and YPRAA solid media and incubated in a 28 °C incubator for 2 days to observe the growth of the transformants.

### 5.7. Subcellular Localization Observation

*A. tumefaciens*-mediated transient expression in *N. benthamiana* was assessed as detailed by Li et al. [30]. The localization signal was observed under a laser confocal microscope (Zeiss, Oberkochen, Germany) 2–3 days after agro-infiltration. Plasmolysis assay of *N. benthamiana* epidermal cells was implemented according to Chen et al. [34] using 0.5 M NaCl.

### 5.8. Western Blotting

Leaves of *N. benthamiana* for observations of subcellular localization were collected, and the total protein was extracted using plant total protein lysis buffer (Sangon Biotech, Shanghai, China), following the manufacturer’s instructions. 0.1 g sample was ground, and 300 μL of lysis buffer was added. According to Li et al. [30], the protein samples were mixed with 2 × loading buffer in equal volumes. Then, the mixture was boiled for 5 min, centrifuged for 10 min, and supernatant was subjected to SDS-PAGE electrophoresis. After transfer of proteins, Ponceau solution staining, blocking, and antibody incubation, protein bands were detected using an Image Quant LAS 4000 mini (GE Healthcare, New York, NY, USA).

### 5.9. cDNA Library Screening

Firstly, we introduced the gene fragment of TcSCP_9014 into the pDHB1 vector. Function detection for the bait vector pDHB1-TcSCP_9014 and cDNA library screening were conducted. pDHB1-TcSCP_9014 was co-transformed with pOst1-NubI and pPR3-N plasmids into yeast cells, respectively. pDHB1-Large T and pDSL-p53 were used as positive control, and pDHB1-Large T and pPR3-N were used as negative control. Then, the mixture was coated onto DDO (SD/- Trp), TDO (SD/- Trp/-Leu), and QDO (SD/- Trp/- Leu/- His) selective media. To improve the efficiency of cDNA library screening, the *His* gene was screened under rigorous conditions, and the concentration of 3-AT was optimized before library screening. 3-AT was added to QDO medium and adjusted to different concentrations of 0 mM, 1 mM, 2.5 mM, 5 mM, 7.5 mM, and 10 mM. Single colony of NYM51 yeast expressing pDHB1-TcSCP_9014 were inoculated into 50 mL of YPDA liquid medium and prepared for competent cells. Then, the transformation of the pPR3-N plasmids was carried out, and the mixture was spread onto QDO screening plates with added 3-AT. The plates were incubated at 30 °C for 3–4 days, and the growth status of the yeast was observed.

Here, the cDNA library was derived from infected wheat spikes. Wheat spikes at the early boot stage were inoculated with *T. controversa*, and infection was confirmed by PCR detection. Infected spike samples were collected once a week until the fourth week to construct the yeast cDNA library. The screening of interacting proteins was performed as described previously [35]. In brief, to 600 μL of competent cells of the NMY51 yeast expressing the bait protein, the following reaction components were added: 7 μg cDNA library plasmids, 100 μL single-strand carrier DNA, and 2.5 mL PEG/LiOAc master mix. The mixture was incubated in a 30 °C water bath for 45 min, and 160 μL of DMSO was subsequently added. Then, the mixture was incubated in a 42 °C water bath for 20 min and collected by centrifugation at 700× *g* for 5 min. Next, 3 mL of 2 × PDA medium solution was added to the culture and incubated in a shaker at 30 °C for 90 min at 150 rpm. Centrifugation at 1000 rpm for 5 min was implemented again to precipitate the culture. NaCl (0.9%) solution was added, and the suspension was coated onto QDO screening plates containing 3-AT. After 3–5 days of cultivation at 30 °C, the colony growth of the plates was observed. Finally, colony PCR assay was performed, and PCR products with a target band of over 1000 bp after electrophoresis were selected and sent for sequencing. Finally, the sequences were submitted to BlastX.

### 5.10. Validation of Interacting Proteins by Yeast Two-Hybrid and BiFC Assays

According to the manufacturer’s instructions for the TIANprep Yeast plasmid DNA kit (TIANGEN, Beijing, China), the plasmids of two interacting proteins in host wheat were extracted. Then, they were transformed to DH5α competent cells, and positive colonies were used to extract the plasmids. Then, the bait plasmids pDHB1-TcSCP_9014 and one kind of prey plasmids of the host were transformed into NYM51 yeast competent cells to observe growth of transformants on QDO/15 mM 3-AT medium. Colonies of transformants were diluted with a 10-fold gradient and spread on QDO/15 mM 3-AT medium to observe their growth.

BiFC assay was applied following Waadt and Kudla [50]. In brief, the encoding genes of the effector TcSCP_9014 and two interacting proteins were fused into p2YC (cYFP) and p2YN (nYFP) vectors. The recombinant plasmids were transformed into *A. tumefaciens*, and *A. tumefaciens* co-infiltration for transient expression was implemented. Fluorescence signals were detected after 2–3 days using a laser scanning confocal microscope (Zeiss, Oberkochen, Germany). The primers are listed in Supplementary Table S1.

## Data Availability

The data presented in this study are available on request from the corresponding author.

## Supplementary Materials

The following supporting information can be downloaded at: https://www.mdpi.com/article/10.3390/plants13111523/s1, Figure S1: Original Western blotting images; Figure S2: Functional detection of bait vector pDHB1-TcSCP_9014; Table S1: Specific primers used in this study; Table S2: Yeast growth on QDO plates for screening of 3-AT concentration.
