# Supplementary material for: Characterization of a Small Cysteine-Rich Secreted Effector, TcSCP_9014, in Tilletia controversa
Source: Plants (Basel). 2024 May 31;13(11):1523. doi: 10.3390/plants13111523 (PMC11174917; doi:10.3390/plants13111523)
Supplement: Supplementary file 1 [file plants-13-01523-s001.zip › plants-2984861-supplementary.pdf]

Supplementary information

Characterization of a small cysteine-rich secreted effector, TcSCP_9014, in *Tilletia controversa*

Zhenzhen Du[1,2], Han Weng[1,2], Huanyu Jia[1], Bin Zhang[1], Boming Wu[2], Wanquan Chen[1], Taiguo Liu[1] and Li Gao[1,3,*]

[1]State Key Laboratory for Biology of Plant Disease and Insect Pests, Institute of Plant Protection, Chinese Academy of Agricultural Sciences, Beijing 100193, P.R. China.

[2]College of Plant Protection, China Agricultural University, Beijing, P.R. 100193, China.

[3]Institute of Plant Protection, Xinjiang Academy of Agricultural Sciences; Key Laboratory of Integrated Pest Management on Crop in Northwestern Oasis, Ministry of China; Scientific Observing and Experimental Station of Korla, Ministry of Agriculture, Xinjiang Urumqi 830091, P.R. China.

Correspondence: lgao@ippcaas.cn

Supplementary Figure S1. Original image of western blotting.

Supplementary Figure S2. Functional detection of bait vector pDHB1-TcSCP_9014

Supplementary Table S1. Specific primers used in this study.

Supplementary Table S2. Yeast growth on QDO plates for screening of 3-AT concentration

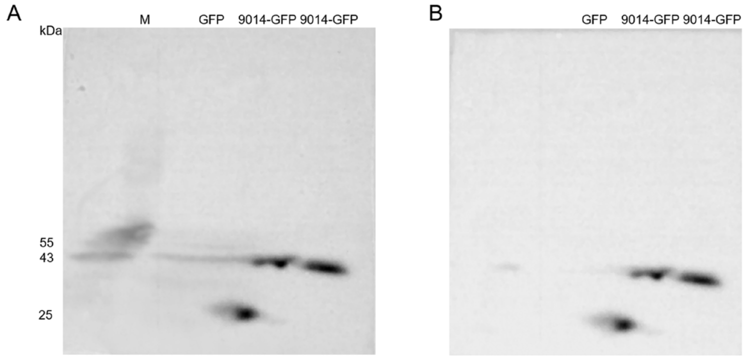

Supplementary Figure S1. Original image of western blotting. (A) Original, uncropped and unadjusted image with marker and protein samples. (B) To better show the GFP and 9014-GFP bands, we also provided the image with protein samples only.

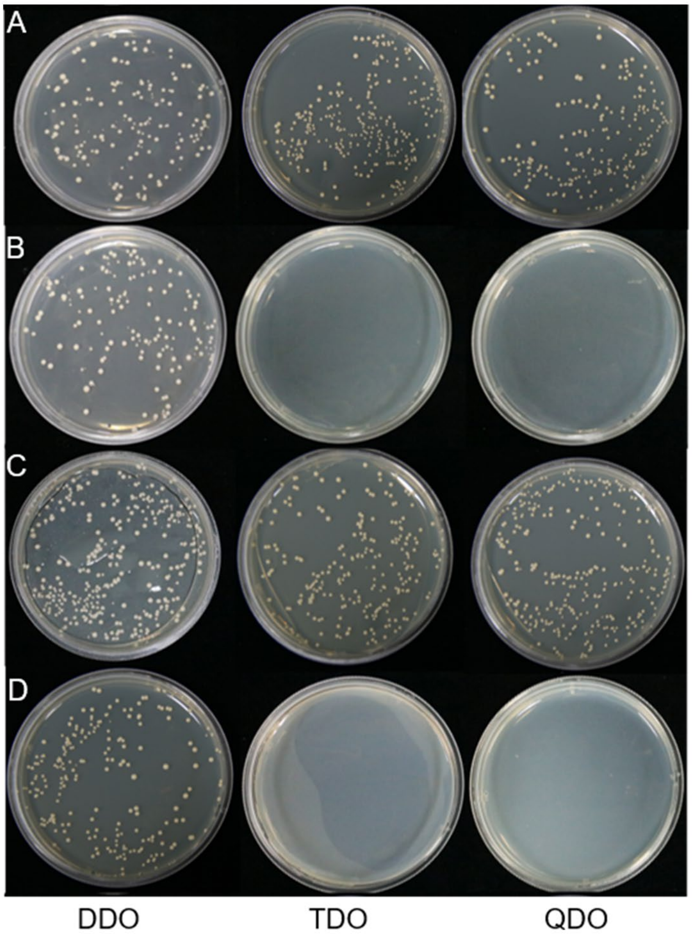

Supplementary Figure S2. Functional detection of bait vector pDHB1-TcSCP_9014. (A) Transformants of positive control group pDHB1-Large T and pDSL-p53 on DDO, TDO and QDO media. (B) Transformants of negative control group pDHB1-Large T and pDSL-p53 on DDO media. (C) Transformants of PDHB1-TcSCP_9014 and pOst1-Nub I on DDO, TDO and QDO media. (D) Transformants of pDHB1-TcSCP_9014 and pPR3-N on DDO media. The

results indicated that the bait vector PDHB1-TcSCP_9014 expressed normally after transformation in yeast and showed no interaction with the empty library plasmids.

Supplementary Table S1. Specific primers used in this study.

	Specific primers for construction of pGR107 recombinant vector
Primers	Sequence (5'-3')
TcSCP_9014-F	TCAGCACCAGCTAGCATCGATATGTCGCACACGATCAACTTTTC
TcSCP_9014-R	AACCGTTCATCGGCGGTCGACCTAGCACGAGATGTCGATGT
Bax-F	TCAGCACCAGCTAGCATCGATATGGACGGGTCCGGGGAG
Bax-*R*	AACCGTTCATCGGCGGTCGACGCCCATCTTCTTCCAGATGG
	Specific primers for construction of pBin-GFP recombinant vector
Primers	Sequence (5'-3')
TcSCP_9014-F	ATTTACGAACGATAGGGTACCATGTCGCACACGATCAACTTTTC
TcSCP_9014-R	GCCCTTGCTCACCATGGATCC GCACGAGATGTCGATGTTG
	Specific primers for construction of pSUC2 recombinant vectors
Primers	Sequence (5'-3')
*9014*SP-F	CGGAATTTTAATTAAGAATTCATGTTCGCCCGCTTCGCC
*9014*SP-R	CACTATAGGGAGAACCTCGAGTGCCAGAGCGGAGGTGGC
9014-F	CGGAATTTTAATTAAGAATTCATGTTCGCCCGCTTCGCC
9014-R	CACTATAGGGAGAACCTCGAGGCACGAGATGTCGATGTTGA
*9014Δ*SP-F	CGGAATTTTAATTAAGAATTCATGTCGCACACGATCAACTTT
*9014Δ*SP-R	CACTATAGGGAGAACCTCGAGGCACGAGATGTCGATGTTGA
	Specific primers for construction of bait vector
Primers	Sequence (5'-3')
TcSCP_9014-F	ATGGCCATTACGGCCATGTCGCACACGATCAACTTTTC
TcSCP_9014-R	ATGGCCGAGGCGGCCGCACGAGATGTCGATGTTG
	Specific primers for construction of p2YC and p2YN recombinant vectors
Primers	Sequence (5'-3')
TcSCP_9014-F	TTTTCGCCAAAAGTTTTAATTAA ATGTCGCACACGATCAACTTT
TcSCP_9014-R	TCATTTGGAGAGGACCTCGAG GCACGAGATGTCGATGTTGA
TaMTAN-F	TCATTTGGAGAGGACCTCGAGATGGCTCCGCCGTCCTCT
TaMTAN-R	TTTTCGCCAAAAGTTTTAATTAAGAGATCAGAGATACATTTGCC
TaGAPDH-F	TCATTTGGAGAGGACCTCGAGATGAAGCCACGTTCTCTCC
TaGAPDH-R	TTTTCGCCAAAAGTTTTAATTAACTTGGTGCTGTGCATGTG

Supplementary Table S2. Yeast growth on QDO plates for screening of 3-AT concentration

3-AT (mM)	The number of clonies growing on QDO plates
0	>150
1	>15
2.5	>15
5	>15
7.5	>15
10	>10
12.5	>5
15	0
